# Integrated Multi-Omics and Molecular Docking Analysis of Malate Degradation by *Lactiplantibacillus plantarum* S4 During Sea Buckthorn Juice Fermentation

**DOI:** 10.3390/foods15193517

**Published:** 2026-10-01

**Authors:** Xiaogang Guo, Menghui Lin, Laifeng Zhuo, Yanru Feng, Thanh Ninh Le, Minjie Zhao, Haiying Cai

**Affiliations:** 1 School of Biological and Chemical Engineering, Zhejiang University of Science & Technology, Hangzhou 310023, China; gxg13709097644@163.com (X.G.); 212403817025@zust.edu.cn (M.L.); 19820234595@163.com (L.Z.); 2 Wushi Yuanmai Agriculture, Forestry and Animal Husbandry Industry Development Co., Ltd., Aksu 843400, China; 17799853284@163.com; 3 College of Biosystems Engineering and Food Science, Zhejiang University, Hangzhou 310058, China; lethanhninh@tuaf.edu.vn (T.N.L.); zhaominjie@zju.edu.cn (M.Z.); 4 Institute of Biotechnology and Food Technology, Thai Nguyen University of Agriculture and Forestry, Thai Nguyen 23000, Vietnam

**Keywords:** sea buckthorn juice, *Lactiplantibacillus plantarum* S4, malic acid degradation, malolactic fermentation, biological deacidification

## Abstract

Sea buckthorn juice (SBJ) is nutritionally valuable, but its high malic acid content contributes to excessive sourness that limits consumer acceptance. In this study, the deacidification phenotypes of two lactic acid bacterial isolates, *Lactiplantibacillus plantarum* S4 and *Lacticaseibacillus paracasei* YJ2, were compared using fermentation phenotyping, sensory evaluation, enzyme assays, untargeted metabolomics, transcriptomics, and molecular docking. After 96 h of fermentation, S4 degraded 91.98% of malic acid, compared with 69.50% for YJ2, and exhibited higher viable-cell counts and overall sensory acceptability under the tested conditions. S4 also showed higher crude-extract malate-conversion activity under MLE-favoring conditions. Molecular docking predicted more favorable interactions of malate with S4-derived MleP and MleS than with the corresponding YJ2 proteins; however, these analyses did not directly measure MleP-mediated transport or MleS-specific catalytic flux. Metabolomic profiling showed lower abundance of malic acid and several other organic acids in S4-fermented SBJ, together with increased abundance of succinic acid, 2-oxoglutaric acid, phenyllactic acid-related metabolites, D-octopine, and N-lactoyl-phenylalanine. Transcriptomic analysis revealed differences between S4 and YJ2 in carbohydrate metabolism, glycolysis, pyruvate metabolism, energy generation, and late-stage cellular maintenance. Integration of these datasets supports a working model in which malate-associated conversion, central-carbon redistribution, organic-acid remodeling, and acid-stress adaptation may contribute to the greater malate-removal phenotype of S4. Because only one isolate of each species was examined, the observed differences should be interpreted as differences between S4 and YJ2 rather than as species-level effects.

## 1. Introduction

Sea buckthorn (*Hippophae rhamnoides* L.) is a valuable resource for functional foods and plant-based beverages because it is rich in bioactive compounds, including ascorbic acid, carotenoids, flavonoids, polyphenols, organic acids, phytosterols, and functional fatty acids [1]. However, raw sea buckthorn juice (SBJ) is characterized by high total acidity and low pH, resulting in sharp sourness, astringency, and sensory irritation, which limit its direct consumption and commercial application. L-Malic acid is a major contributor to this pronounced acidic perception. Therefore, developing mild deacidification strategies that preserve the nutritional and functional attributes of SBJ while improving its flavor quality is essential for its value-added processing.

Lactic acid bacteria (LAB) fermentation is a mild and effective bioprocessing strategy for improving the quality of fruit and vegetable juices. Through the metabolism of organic acids, sugars, amino acids, phenolic compounds, and volatile flavor compounds, LAB fermentation can modulate acidity, enhance mouthfeel, improve nutritional composition, and increase storage stability [2,3,4]. *Lactiplantibacillus plantarum* (*L. plantarum*) and *Lacticaseibacillus paracasei* (*L. paracasei*) are widely used in non-dairy fruit and vegetable juice fermentation because of their acid-stress tolerance, substrate adaptability, and metabolic versatility [5]. Previous studies have shown that different LAB strains can alter viable cell counts, reducing sugar content, titratable acidity, malic acid concentration, volatile profiles, and sensory characteristics in SBJ. Nuclear magnetic resonance (NMR)- and gas chromatography-ion mobility spectrometry (GC-IMS)-based analyses have further demonstrated that *L. plantarum* promotes the consumption of L-malic acid, certain amino acids, and nucleosides while reshaping multiple classes of volatile flavor compounds. These findings indicate that LAB fermentation of SBJ involves not only deacidification but also extensive metabolic and flavor remodeling [6,7,8].

Malolactic fermentation (MLF) is a key biological deacidification pathway of LAB. This process is generally catalyzed by malolactic enzyme (MLE), which decarboxylates dicarboxylic L-malic acid into monocarboxylic L-lactic acid with the release of CO_2_, thereby reducing the proportion of strongly irritating acids and improving taste balance [9,10]. However, malic acid degradation is not a single-step reaction. It may involve malate transport, MLE-mediated decarboxylation, pyruvate formation, lactate dehydrogenase regulation, coordinated citrate metabolism, and the remodeling of dicarboxylic acid- and tricarboxylic acid (TCA)-related metabolic nodes [9,11]. The MAE-associated malate–pyruvate pathway and the MDH-associated malate–oxaloacetate pathway were likely functioned as an auxiliary route for carbon flux redistribution in LAB deacidification. MAE catalyzes the oxidative decarboxylation of malate to pyruvate and CO_2_, thereby linking malate degradation with pyruvate metabolism, redox balance, and downstream metabolite formation. MDH catalyzes the reversible interconversion between malate and oxaloacetate and participates in the regulation of dicarboxylic acid metabolism and TCA-related nodes.

Moreover, the efficiency of malic acid degradation by LAB is highly strain-dependent. Even under identical fermentation conditions, different strains may exhibit distinct growth patterns, organic acid utilization capacities, acid-stress responses, and metabolic outputs [12,13,14]. These differences may be associated with malate transport, substrate recognition by key enzymes, intracellular pH regulation, energy metabolism, membrane lipid remodeling, and protein quality-control systems. *L. plantarum* can maintain cellular homeostasis under acid stress by regulating ATPase activity, amino acid metabolism, membrane lipid composition, transport systems, and stress-related genes, including dnaK, groEL, groES, hrcA, and clpE [15,16,17]. Nevertheless, these adaptive mechanisms do not fully explain the strain-specific differences in malic acid degradation efficiency in SBJ.

Therefore, the present study aimed to compare the malic acid degradation phenotypes of *L. plantarum* S4 and *L. paracasei* YJ2 under the same highly acidic SBJ fermentation conditions and to identify biochemical and molecular features associated with their different malate-removal capacities. Preliminary observations indicated that S4 removed a substantially greater proportion of malic acid than YJ2 during 96 h of fermentation. To investigate the biological processes associated with this difference, fermentation phenotyping, enzyme assays, untargeted metabolomics, transcriptomics, and comparative molecular docking were integrated. Rather than establishing species-level differences or definitive pathway flux, the study was designed to generate an integrated working model of the biochemical and cellular processes associated with the contrasting phenotypes of these two isolates. The findings may provide a basis for subsequent validation using additional strains and targeted functional experiments.

## 2. Materials and Methods

### 2.1. Materials and Strains

SBJ was obtained from Xinjiang Yuanmai Trading Co., Ltd. (Aksu, China). *Lactiplantibacillus plantarum* S4 was deposited in the China General Microbiological Culture Collection Center under accession number CGMCC No. 37352. *Lacticaseibacillus paracasei* YJ2 was preserved in our laboratory. Sodium hydroxide (AR), potassium hydrogen phthalate (≥99.8%), phenolphthalein, HEPES, L-malic acid, β-nicotinamide adenine dinucleotide (NAD^+^), manganese chloride (MnCl_2_), and absolute ethanol were purchased from Aladdin Chemical Reagent Co., Ltd. (Shanghai, China). The MRS medium was used for strain activation and cultivation. A commercial kits for malic acid determination was purchased from Beyotime Biotechnology Co., Ltd. (Shanghai, China). Malic enzyme (MAE) and malate dehydrogenase (MDH) activity kits were obtained from Aidi Biotechnology Co., Ltd. (Qingdao, China) and Suzhou Grace Biotechnology Co., Ltd. (Suzhou, China), respectively.

### 2.2. Experimental Design and Fermentation Procedure

Fresh SBJ was diluted with sterile water at a ratio of 1:1, supplemented with 6% glucose to adjust the soluble solid content, pasteurized at 65 °C for 30 min, and cooled to room temperature.

For strain activation, S4 and YJ2 were separately inoculated into MRS broth and anaerobically cultured at 35 °C for 8–12 h. Each strain was subcultured twice with a 5% inoculum (*v*/*v*) to obtain cells in the logarithmic growth phase. The bacterial suspensions were adjusted with phosphate-buffered saline (PBS) to an OD600 of 1.0, corresponding to approximately 10^8^–10^9^ colony-forming units (CFU)/mL.

The treated SBJ was inoculated with 2% activated bacterial suspension (*v*/*v*), sealed, and anaerobically fermented at 35 °C for 96 h. The same independent fermentation vessels were sampled longitudinally at 0, 24, 48, 72, and 96 h; 2 mL of fermented juice was collected at each time point, centrifuged at 10,000 rpm at 4 °C for 10 min, and filtered through a 0.22 μm aqueous membrane. The filtrates were stored for subsequent analyses.

### 2.3. Physicochemical Analysis and Sensory Evaluation

The pH, titratable acidity, viable cell counts, and soluble solid content were determined according to the methods of Guo et al. and Lugo-Zarate et al. [18,19]. The concentration of L-malic acid was quantified with a commercial assay kit purchased from Beyotime Biotechnology (Shanghai, China), based on the oxidation of L-malic acid to oxaloacetic acid by malate dehydrogenase, during which NAD^+^ is reduced to NADH. The generated NADH reduces WST-8 to orange formazan in the presence of the electron-coupling reagent 1-methoxy-5-methylphenazinium methyl sulfate, and the absorbance at 450 nm is proportional to the L-malic acid content.

The physicochemical analyses were conducted using n = 3 independent fermentation replicates for each strain and time point. For each biological replicate, each analytical determination was performed in triplicate, and technical replicates were averaged before statistical analysis.

For the enzyme assays, crude enzyme extracts were independently prepared from n = 3 biological fermentation replicates collected at 48 h. Each crude extract was assayed in triplicate. Technical replicates were averaged to yield a single value for each independent biological replicate; therefore, the biological replicate, rather than the individual technical measurement, was used as the statistical unit.

Crude-extract malate-conversion activity under MLE-favoring conditions was evaluated using a substrate-depletion assay. Bacterial cells were harvested by centrifugation at 10,000× *g* for 10 min at 4 °C, washed twice with phosphate buffer (pH 6.0), and resuspended in the same buffer. The cells were disrupted by ultrasonication in an ice bath, followed by centrifugation at 12,000× *g* for 15 min at 4 °C. The resulting supernatant was collected as the crude enzyme extract. The enzyme assay was performed in a total reaction volume of 1.0 mL containing phosphate buffer (pH 6.0), L-malic acid (100 μg/mL), MnCl_2_ (63.5 μg/mL), NAD^+^ (132.7 μg/mL), and an appropriate volume of crude enzyme extract. After pre-incubation at 30 °C for 5 min, the reaction was initiated by the addition of the crude enzyme extract and allowed to proceed at 30 °C for 5 min. The reaction was terminated by adding an equal volume of ice-cold perchloric acid, followed by centrifugation at 12,000× *g* for 10 min. The residual L-malic acid in the supernatant was subsequently quantified. Apparent malate-conversion activity was calculated from the decrease in L-malic acid concentration before and after the reaction. One unit (U) was defined as the activity corresponding to consumption of 1 μmol of L-malic acid per minute under these assay conditions [20,21]. Because the assay used a crude cell extract and quantified net malate disappearance, the signal cannot be assigned exclusively to MleS; MAE and MDH may also contribute to changes in malate concentration. malic-lactic enzyme activity has been reported and interpreted as the malic acid conversion (apparent MLE-related) activity in crude extracts, not as MleP transport activity or purified MleS-specific activity. Because the assay was performed using crude cell extracts, the measured activity represents the overall malate-conversion capacity of the extract rather than a protein-normalized specific activity. Activities were expressed per milliliter of crude extract in the present study because total protein concentration and harvested biomass normalization were not determined. Therefore, comparisons between strains should be interpreted as differences in crude-extract malate-conversion capacity under the defined assay conditions, rather than differences in intrinsic enzyme catalytic efficiency.

MAE activity was determined based on its catalysis of malate oxidative decarboxylation to pyruvate and CO_2_, accompanied by NAD(P)^+^ reduction. Enzyme activity was calculated from the increase in NADH absorbance at 340 nm. MDH activity was measured based on the MDH-catalyzed reduction of oxaloacetate to malate using NADH as the electron donor, and activity was calculated from the decrease in absorbance at 340 nm.

During the sensory evaluation phase, 10 professionally trained evaluators with experience in sensory evaluation assessed the fermented SBJ samples. Before evaluation, all samples were assigned random three-digit codes to minimize identification bias. The samples were presented in a randomized order and served under controlled conditions (20 mL per sample at 25 °C). Assessors were instructed to rinse their mouths with pure water between evaluations to minimize sensory carry-over effects. The assessors were blinded to the specific sample identity and fermentation treatment.

The sensory evaluation included three independent fermentation replicates for each treatment. Each biological replicate was evaluated separately, and the scores from the 10 assessors were averaged to obtain the sensory score for each biological replicate. The mean value of the three biological replicates was subsequently used for statistical analysis. See Table 1 for sensory evaluation criteria.

All fermentation experiments were performed using n = 3 independent biological replicates. Each biological replicate consisted of an independently prepared fermentation vessel inoculated with a separately prepared bacterial culture. Unless otherwise specified, analytical measurements were performed in triplicate for each biological replicate, and the mean of the technical measurements was treated as one biological observation.

In parallel with the inoculated groups, a time-matched uninoculated SBJ control was prepared from the same batch of treated juice without addition of bacterial culture and was incubated under the same conditions (35 °C, anaerobic conditions) for 96 h. This control was used to distinguish bacterial fermentation-associated changes from physicochemical changes caused by incubation alone. The uninoculated control was sampled at 0, 24, 48, 72, and 96 h using the same procedure as the inoculated groups. Thus, changes associated with bacterial fermentation could be distinguished from those resulting from incubation of the SBJ matrix itself.

### 2.4. Untargeted Metabolomic Analysis of Fermented SBJ

Fermentation samples were collected at 96 h, including the time-matched uninoculated control (B1), the *Lactiplantibacillus plantarum* S4 fermentation group, and the *Lacticaseibacillus paracasei* YJ2 fermentation group, with six biological replicates for each group. A total of 18 biological samples were subjected to untargeted LC–MS/MS analysis.

For metabolite extraction, 200 μL of each sample was mixed with 800 μL of extraction solvent consisting of methanol and acetonitrile (1:1, *v*/*v*) containing four internal standards, including L-2-chlorophenylalanine (0.02 mg/mL). After vortex mixing for 30 s, the samples were subjected to low-temperature ultrasonication for 30 min at 5 °C and 40 kHz, incubated at −20 °C for 30 min, and centrifuged at 13,000× *g* for 15 min at 4 °C. The supernatant was dried under nitrogen and reconstituted in 120 μL of acetonitrile/water (1:1, *v*/*v*). After vortex mixing and ultrasonication for 5 min at 5 °C, the samples were centrifuged at 13,000× *g* for 10 min at 4 °C, and the resulting supernatants were transferred to autosampler vials for LC–MS/MS analysis.

LC–MS/MS analysis was performed using a Vanquish UHPLC system coupled to a Q-Exactive HF mass spectrometer (Thermo Scientific, Bremen, Germany). Chromatographic separation was performed using an ACQUITY UPLC HSS T3 column (100 mm × 2.1 mm i.d., 1.8 μm; Waters, Milford, MA, USA). Mobile phase A consisted of water/acetonitrile (95:5, *v*/*v*) containing 0.1% formic acid, whereas mobile phase B consisted of acetonitrile/isopropanol/water (47.5:47.5:5, *v*/*v*/*v*) containing 0.1% formic acid. The injection volume was 3 μL and the column temperature was maintained at 40 °C.

The mass spectrometer was operated with an electrospray ionization source in both positive- and negative-ion modes. The acquisition mass range was *m*/*z* 70–1050. The sheath-gas and auxiliary-gas flow rates were set to 50 and 13 arb, respectively. The heater temperature and capillary temperature were maintained at 425 and 325 °C, respectively. The spray voltages were +3500 V in positive-ion mode and −3200 V in negative-ion mode, and the S-lens RF level was set to 50. Stepped normalized collision energies of 20, 40, and 60% were used for MS/MS acquisition. The mass resolutions were 60,000 for full MS and 15,000 for MS/MS.

A pooled quality-control (QC) sample was prepared by mixing equal aliquots of extracts from all analytical samples and was processed in the same manner as the experimental samples. One QC sample was injected after every 5–15 analytical samples throughout the analytical sequence to monitor the stability and reproducibility of the LC–MS/MS system. Raw LC–MS/MS data were processed using Progenesis QI v3.0 (Waters Corporation, Milford, MA, USA) for baseline filtering, peak detection, integration, retention-time correction, and peak alignment. The resulting data matrix contained retention time, *m*/*z*, peak intensity, and metabolite-annotation information. Metabolite identification was performed by matching MS and MS/MS information against HMDB, METLIN, and an in-house database. The mass error used for MS-based metabolite annotation was restricted to <10 ppm, and MS/MS spectral-matching scores were additionally used to support metabolite identification.

Features detected in at least 80% of the samples within at least one experimental group were retained. Missing values were imputed using the minimum observed value in the data matrix, and peak intensities were normalized by sum normalization. Features showing a relative standard deviation (RSD) > 30% in the QC samples were excluded, and the retained data were log10-transformed before subsequent statistical analysis.

Prior to multivariate statistical analysis, the data were subjected to Pareto scaling. Principal component analysis (PCA) and partial least-squares discriminant analysis (PLS-DA) were performed to evaluate global differences in metabolite profiles among the B1, S4, and YJ2 groups. A 95% confidence level was used for visualization of the PLS-DA score plot, and 200 permutation tests were performed to evaluate model robustness and potential overfitting.

Differential metabolites were defined as metabolites with a variable importance in projection (VIP) value > 1 and a Student’s *t*-test *p* value < 0.05. No separate fold-change threshold was applied. Differential metabolites were subsequently subjected to Kyoto Encyclopedia of Genes and Genomes (KEGG) pathway annotation and enrichment analysis, and hierarchical clustering was used to visualize relative metabolite-abundance patterns.

### 2.5. Transcriptomic Analysis of Fermented SBJ

Fermentation broths of *Lactiplantibacillus plantarum* S4 and *Lacticaseibacillus paracasei* YJ2 were collected at 48 and 96 h of fermentation. Four experimental groups were included (S4_48 h, S4_96 h, YJ2_48 h, and YJ2_96 h), with three independent biological fermentation replicates for each group (n = 3). Each biological replicate was obtained from an independently prepared fermentation vessel.

Samples were centrifuged at 8000 rpm at 4 °C for 5 min, and the resulting bacterial pellets were immediately frozen in liquid nitrogen and stored until RNA extraction. Total RNA was extracted using the cetyltrimethylammonium bromide (CTAB) method, followed by removal of genomic DNA. Ribosomal RNA was depleted using the RiboCop rRNA Depletion Kit for Mixed Bacterial Samples (Lexogen, Inc., Greenland, NH, USA). The remaining RNA was randomly fragmented to approximately 300 bp and reverse-transcribed into double-stranded cDNA using random primers. During second-strand cDNA synthesis, dUTP was incorporated instead of dTTP. After end repair, 5′ phosphorylation, 3′ A-tailing, and adapter ligation, the dUTP-containing second strand was removed using UNG enzyme to generate strand-specific libraries. Libraries were prepared using the Illumina Stranded mRNA Prep, Ligation Kit (Illumina, San Diego, CA, USA), and paired-end sequencing was performed on an Illumina NovaSeq X Plus platform.

Raw sequencing reads were quality-filtered using fastp v0.23.4. Clean reads from the two strains were mapped independently to their corresponding reference sequences using Bowtie2 v2.5.4. For S4, reads were mapped against the *Lactiplantibacillus plantarum* ZJ316 reference genome (RefSeq assembly GCF_000338115.2; GenBank assembly GCA_000338115.2). ZJ316 was selected because it represents a high-quality complete genome assembly with comprehensive annotation, providing a reliable reference for transcriptomic alignment and gene-level quantification of *L. plantarum* S4. For YJ2, reads were mapped against the complete genome assembly of *Lacticaseibacillus paracasei* ATCC 334 (RefSeq assembly GCF_000014525.1; GenBank assembly GCA_000014525.1). ATCC 334 was selected as a reference genome because it represents a well-characterized, completely sequenced, and well-annotated genome of *L. paracasei*, providing a reliable reference for transcriptomic alignment and gene-level quantification. The genome mapping ratios were evaluated after alignment. The mapping ratios were 48.15–51.65% for S4_48 h, 44.11–54.83% for S4_96 h, 73.78–78.09% for YJ2_48 h, and 37.92–47.15% for YJ2_96 h, respectively. Gene-level expression was quantified using RSEM v1.3.3, and transcripts per million (TPM) values were generated to describe relative transcript abundance and for expression-profile visualization.

Because S4 and YJ2 belong to different bacterial species, cross-strain expression comparisons were restricted to homologous genes within a defined orthology framework. Protein-coding sequences from the two reference genomes were compared bidirectionally using DIAMOND v2.1.9. To minimize the influence of gene duplication and non-equivalent homologous relationships, only genes showing unique reciprocal one-to-one orthologous relationships were retained for S4-versus-YJ2 comparative transcriptomic analysis. The orthology filtering step was used to define comparable gene pairs between the two strains.

Differential-expression analysis was performed using DESeq2 v1.42.0. Genes showing an absolute log2 fold change of at least 1 and a Benjamini–Hochberg-adjusted *p* value (padj) < 0.05 were considered significantly differentially expressed. Multiple-testing correction was performed using the Benjamini–Hochberg procedure. For cross-strain comparisons, log2FC values were calculated only for genes retained in the one-to-one orthologue dataset.

To directly evaluate temporal transcriptional changes within each strain independently of inter-strain differences, additional within-strain differential-expression analyses were performed by comparing S4 at 48 h with S4 at 96 h (S4_48 h vs. S4_96 h) and YJ2 at 48 h with YJ2 at 96 h (YJ2_48 h vs. YJ2_96 h). These analyses were performed using three biological replicates at each time point and the same differential-expression criteria described above.

Gene Ontology (GO) enrichment analysis was performed using GOATOOLS v1.4.4 with the GO database version 2026.0211. Functional annotation was additionally supported by eggNOG v2020.06, and Kyoto Encyclopedia of Genes and Genomes (KEGG) pathway annotation and enrichment analyses were performed using the KEGG database version 2026.02. Enrichment results were interpreted on the basis of significantly differentially expressed genes identified in the corresponding comparison.

### 2.6. Homology Modeling and Molecular Docking

Three-dimensional models of MleP, MleS, MAE, and MDH from *Lactiplantibacillus plantarum* S4 and *Lacticaseibacillus paracasei* YJ2 were generated using SWISS-MODEL. Template selection was based on sequence identity, sequence coverage, and model-quality estimates provided by SWISS-MODEL. The templates, sequence identities, sequence coverages, Global Model Quality Estimate (GMQE) values and predicted oligomeric states of the models used for subsequent docking analysis are summarized in Table 2.

The three-dimensional structure of L-malic acid was obtained from PubChem. Receptor and ligand structures were prepared using AutoDockTools 1.5.6. Polar hydrogen atoms were added and Gasteiger charges were assigned during PDBQT preparation. No additional pH-specific optimization of the protonation state was performed [22,23].

Molecular docking was performed using AutoDock Vina 1.1.2 with a global/blind docking strategy. For each protein model, the docking search space was defined using a grid box of 70 × 70 × 70 Å with a grid center of −0.559, 0.401, 0.115. The same box center was initially applied to all protein models to maintain consistent computational conditions for comparative docking analysis; however, the large grid dimensions allowed exploration of the entire protein surface and potential ligand-binding regions.

The default random seed implemented in AutoDock Vina was used. Each docking calculation was performed with three independent runs using random seed setting to evaluate docking reproducibility. The top-ranked poses were initially ranked according to predicted binding affinity (kcal/mol), and the representative pose was selected based on the lowest predicted binding energy together with reasonable ligand placement within the predicted binding region and interaction pattern with surrounding residues.

Docking results were visualized using PyMOL 3.1.3 (Schrödinger, LLC, New York, NY, USA).

For MleS, NAD^+^ and Mn^2+^ were not explicitly incorporated into the docking model. Because these cofactors are relevant to the biochemical function of malolactic enzyme, the present docking model does not represent the complete catalytically competent MleS complex. Accordingly, the MleS docking results were used only for comparative assessment of potential malate-recognition patterns under the same computational conditions and were not interpreted as quantitative measures of physiological binding affinity or catalytic efficiency. Similarly, docking of malate to MleP was used only to examine possible differences in substrate recognition and not to infer transmembrane transport rates.

### 2.7. Statistical Analysis

Statistical analyses were performed using GraphPad Prism 8 (GraphPad Software Inc., San Diego, CA, USA). Data are presented as mean ± standard deviation (SD), unless otherwise indicated. Technical replicate measurements were averaged before statistical analysis, and independent biological replicates were used as the experimental units.

For physicochemical parameters measured over the fermentation period, treatment, fermentation time, and their interaction were evaluated using two-way ANOVA, followed by Tukey-adjusted multiple comparisons. The statistical model was selected according to the experimental structure: when the same independent fermentation replicate was sampled repeatedly over time, fermentation replicate was treated as the repeated/random factor.

Enzyme activities at 48 h were compared between S4 and YJ2 using an unpaired two-tailed Student’s *t*-test based on n = 3 independent biological replicates.

For sensory data, each sensory attribute was analyzed separately using a linear mixed-effects model, with sample treatment as a fixed effect and assessor as a random effect. Pairwise comparisons among samples were adjusted using Tukey’s method. Statistical significance was defined as *p* < 0.05.

## 3. Results and Discussion

### 3.1. Fermentation Performance, Malic Acid Degradation, and Sensory Changes During SBJ Fermentation

**Figure 1:**
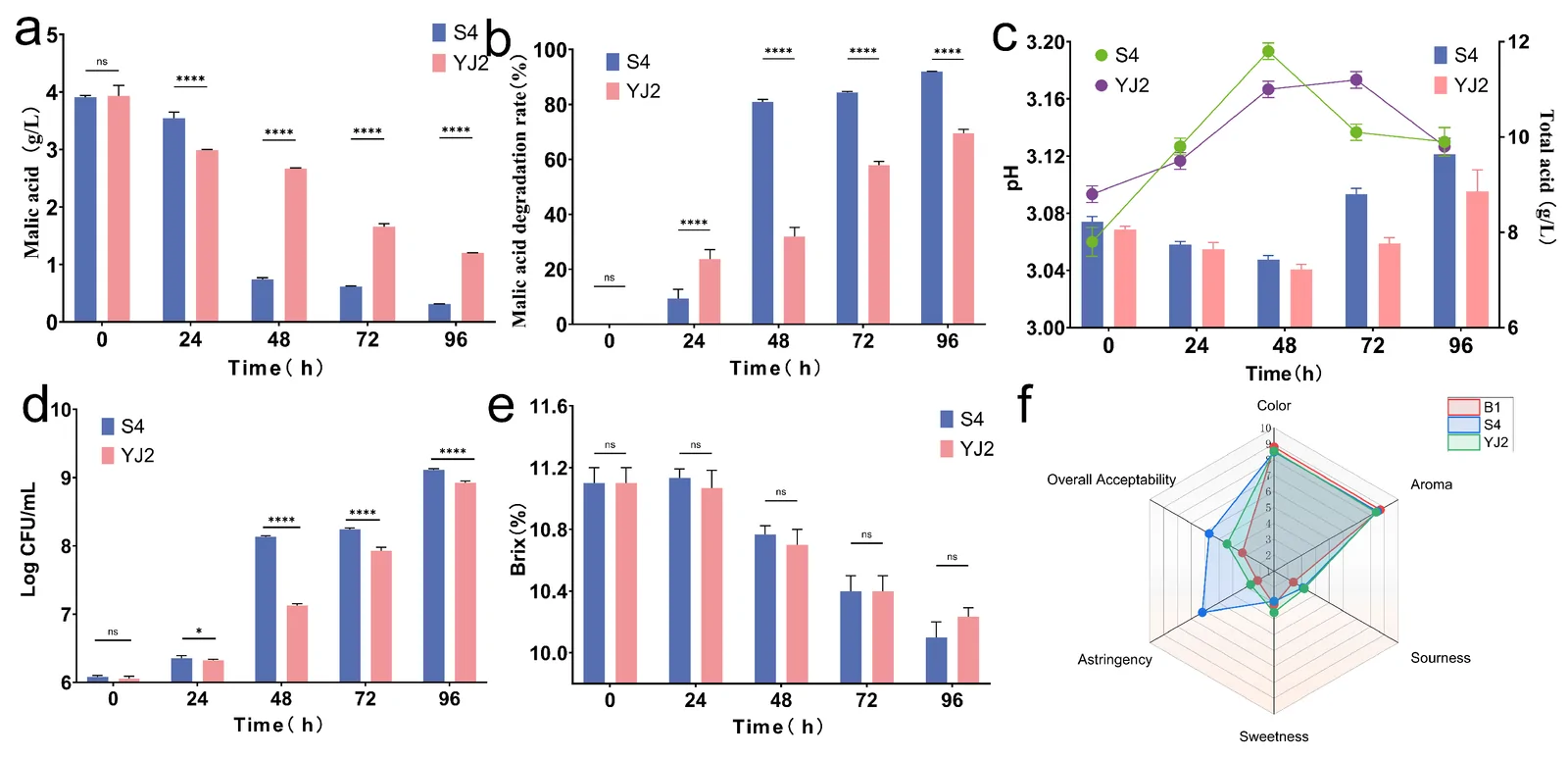
Malic acid degradation dynamics and fermentation quality changes in sea buckthorn juice fermented by LAB strains. (**a**) Malic acid content; (**b**) malic acid degradation rate; (**c**) pH and titratable acidity; (**d**) viable cell counts; (**e**) soluble solid content; and (**f**) sensory profiles. S4, *Lactiplantibacillus plantarum* S4; YJ2, *Lacticaseibacillus paracasei* YJ2; B1, 96-h time-matched uninoculated sea buckthorn juice control. Data are presented as mean ± SD. ns, not significant; * *p* < 0.05, **** *p* < 0.0001.

During SBJ fermentation, both S4 and YJ2 survived and proliferated in the highly acidic plant-based matrix, but they differed markedly in growth kinetics, malic acid utilization, acidity modulation, and sensory improvement. The changes in malic acid further demonstrated a clear difference in deacidification capacity between S4 and YJ2 under the tested fermentation conditions (Figure 1a,b). Initially, the malic acid concentration in both groups was approximately 3.9 g/L. During fermentation, malic acid decreased much faster in the S4 group than in the YJ2 group, especially between 24 and 48 h. At 96 h, the residual malic acid level decreased to approximately 0.31 g/L in the S4 group, whereas approximately 1.19 g/L remained in the YJ2 group. The malic acid degradation rate reached 91.98% in S4, compared with 69.50% in YJ2. These results provide direct phenotypic evidence that S4 possessed stronger malate deacidification capacity. Malolactic fermentation (MLF) converts dicarboxylic L-malic acid into monocarboxylic L-lactic acid with the release of CO_2_, thereby reducing acid irritation and improving taste balance [9]. Previous studies have also shown that *L. plantarum*-mediated MLF can rapidly consume L-malic acid, amino acids, and nucleosides and reshape the metabolic profile of SBJ [7].

Changes in pH and total acidity are shown in Figure 1c. In the S4 group, pH increased rapidly during the first 48 h and reached its maximum at 48 h, whereas YJ2 showed a slower increase and a delayed pH peak. This pattern was consistent with the faster depletion of malic acid in the S4 group. In contrast, total acidity decreased during the early fermentation stage and increased again at the late stage. Notably, S4 showed higher final total acidity than YJ2 despite its lower residual malic acid content. These results indicate that fermentation did not simply reduce total acidity. Rather, S4 selectively decreased malic acid while modifying the overall acid composition of SBJ. Viable cell counts showed that both strains survived and proliferated in the acidic SBJ matrix (Figure 1d). The two groups exhibited comparable cell counts at 0 and 24 h, although a slight but significant difference was observed at 24 h. From 48 h onward, the viable cell count of S4 was significantly higher than that of YJ2. At 96 h, S4 reached 9.1 log CFU/mL, exceeding the level observed for YJ2. These results suggest that S4 had better growth performance in SBJ, which may have supported its higher malic acid degradation efficiency.

During fermentation, the soluble solid content decreased gradually in both groups (Figure 1e). However, no significant difference in °Brix was observed between S4 and YJ2 at any sampling point, indicating comparable overall consumption of soluble solids.

Sensory evaluation results further revealed differences among the 96-h time-matched uninoculated control, S4-fermented SBJ, and YJ2-fermented SBJ groups (Figure 1f). The three samples showed similar color and aroma scores, whereas clear differences were observed in sourness, astringency, sweetness, and overall acceptability. Compared with the 96-h time-matched uninoculated control, fermented samples showed reduced sourness and astringency. Among them, S4-fermented SBJ exhibited the highest overall acceptability, indicating that S4 fermentation more effectively improved the sensory quality of SBJ. Overall, it was demonstrated that S4 displayed better malic acid degradation efficiency, growth performance, c acid-profile modulation, and sensory acceptability during SBJ fermentation compared to YJ2.

### 3.2. Enzyme Activities and Comparative Docking of Malate-Associated Proteins

S4 exhibited stronger malic acid degradation and deacidification capacity than YJ2 during SBJ fermentation. In LAB, L-malic acid can be metabolized through multiple routes, including the MleP/MleS-mediated malolactic fermentation pathway, the MAE-associated malate–pyruvate branch, and the MDH-associated malate–oxaloacetate/dicarboxylic acid branch.

**Figure 2:**
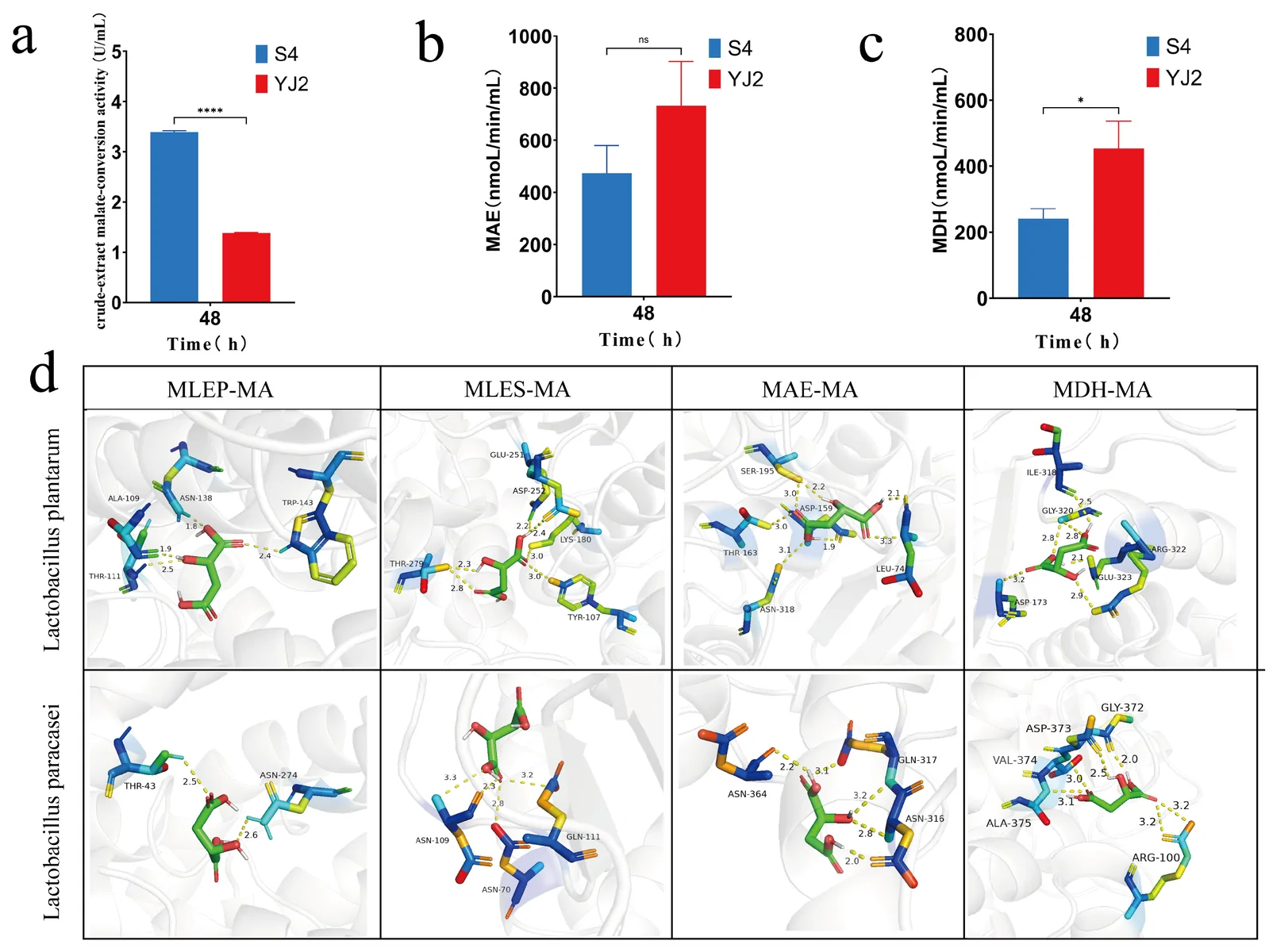
(**a**) Crude-extract malate-conversion activity under MLE-favoring assay conditions; (**b**) malic enzyme (MAE) activity; (**c**) malate dehydrogenase (MDH) activity; (**d**) representative molecular docking conformations of malic acid with MleP, MleS, MAE, and MDH from S4 and YJ2. Data are presented as mean ± SD. ns, not significant; * *p* < 0.05; **** *p* < 0.0001.

As shown in Figure 2a, S4 exhibited significantly higher crude-extract malate-conversion activity than YJ2 at 48 h. This substrate-depletion assay was performed with a cell-free crude extract and therefore does not measure membrane transport through MleP. In addition, because net malate disappearance can reflect the activity of more than one malate-metabolizing enzyme, the Figure 2a signal is interpreted as apparent MLE-associated malate-conversion activity rather than MleS-specific activity. The separately measured MAE and MDH activities were lower in S4 than in YJ2 (Figure 2b,c). indicating that these two measured branches alone are unlikely to account for the greater malate depletion observed in S4. Together with the crude-extract malate-conversion assay and docking results, this pattern is consistent with a possible contribution of the MleP–MleS pathway to the different malate-removal phenotypes between S4 and YJ2. However, because crude-extract activity was not normalized by protein content or biomass, the assay does not directly quantify pathway-specific catalytic capacity.

Molecular docking between malic acid and four malate-related proteins, namely MleP, MleS, MAE, and MDH (Figure 2), further suggested a potential structural basis for strain-dependent differences in this pathway. As summarized in Table 3, S4-MleP displayed lower binding energy with malic acid than YJ2-MleP, suggesting a more favorable predicted interaction with malic acid. However, docking evaluates protein–ligand binding rather than transmembrane transport kinetics; the MleP result therefore provides structural support for a possible difference in substrate recognition, not direct evidence of enhanced malate uptake. S4-MleS likewise showed lower binding energy with malic acid than YJ2-MleS, indicating a more favorable predicted enzyme–substrate interaction during the catalytic step. Representative docking conformations further showed that malic acid was more deeply embedded in the predicted binding pockets of S4-MleP and S4-MleS, whereas the corresponding sites in YJ2 appeared relatively shallow or open. Together with the phenotypic malate-depletion data and the crude-extract activity assay, these structural observations are consistent with a contribution of the MleP-MleS pathway to the faster malate removal by S4, while the relative transport capacity of MleP remains to be verified experimentally.

Moreover, the binding energies of malic acid based on molecular docking between S4-MAE and YJ2-MAE proteins was similar. YJ2-MDH had a slightly stronger binding tendency toward malic acid than S4-MDH.

These results suggest that MAE and MDH may participate in malate-associated metabolic processes linked to pyruvate metabolism. This interpretation is consistent with the observed changes in multiple pyruvate- and fermentation-related metabolites, including the accumulation of lactate- and pyruvate-derived compounds such as phenyllactic acid derivatives, hydroxyphenyllactic acid derivatives, D-octopine, and N-lactoyl-phenylalanine. However, the present metabolomic data do not establish direct carbon transfer from malate into these products.

The higher MDH activity observed in YJ2 may reflect a compensatory response to residual malic acid stress, or a greater reliance on dicarboxylic acid interconversion when malate processing via the MleP/MleS-associated pathway is less favorable. In this context, MDH likely contributes primarily to malate–oxaloacetate balance, organic acid homeostasis, and acid-stress buffering, rather than directly driving malic acid removal.

### 3.3. Untargeted Metabolomics Reveals Malic Acid Depletion and Metabolic Redistribution

**Figure 3:**
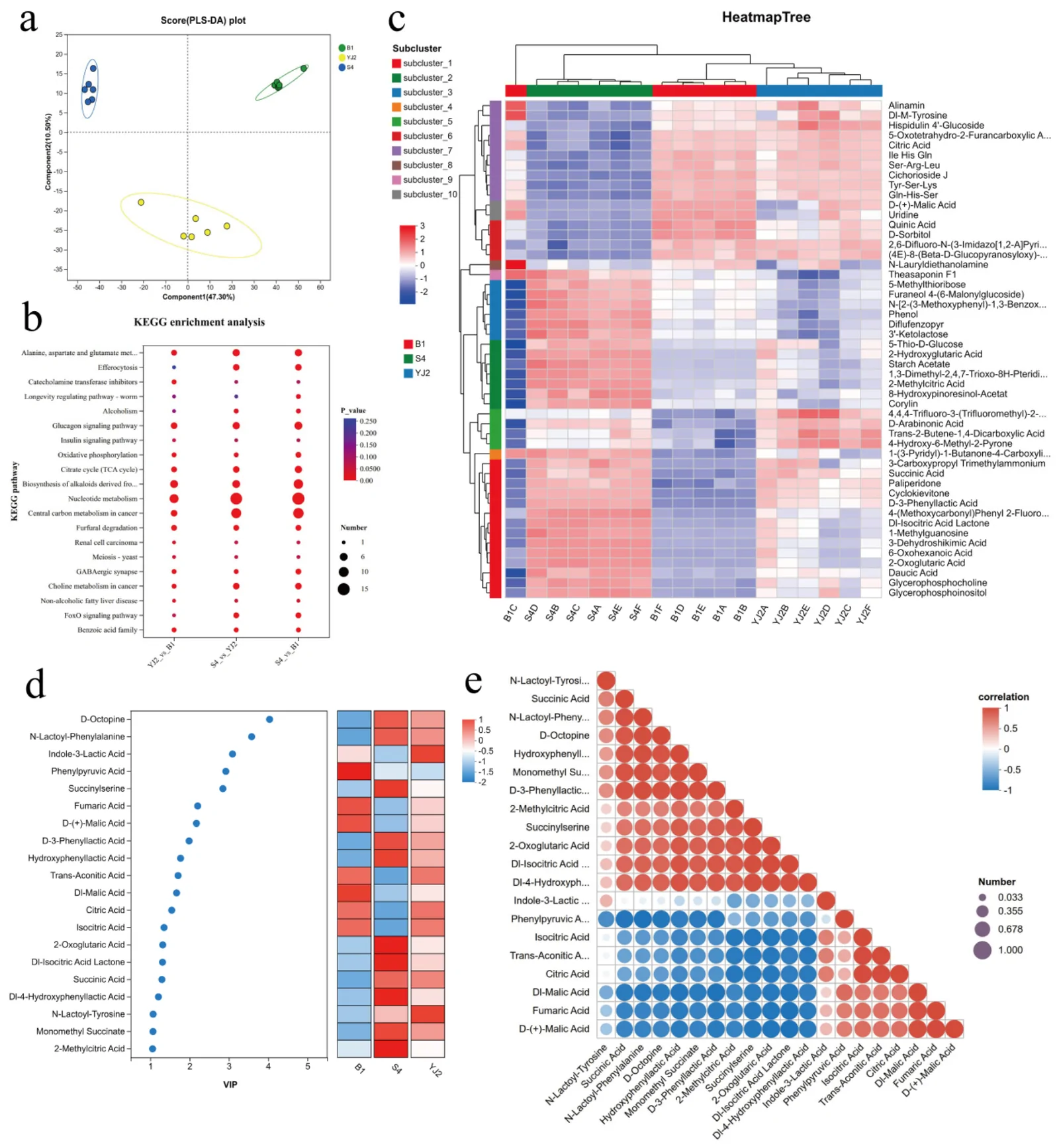
Untargeted metabolomics reveals malic acid depletion and extensive metabolic remodeling during SBJ fermentation. (**a**) PLS-DA score plot of B1, S4, and YJ2 samples; (**b**) KEGG enrichment analysis of differential metabolites; (**c**) hierarchical clustering heatmap of representative differential metabolites; (**d**) VIP analysis and relative-abundance heatmap of key metabolites associated with organic-acid and fermentation-related metabolism; and (**e**) correlation analysis of selected metabolites. The PLS-DA model yielded R^2^X(cum) = 0.589, R^2^Y(cum) = 0.967, and Q^2^(cum) = 0.903. Model robustness was evaluated using 200 permutation tests, and none of the permuted models exceeded the original model for either R^2^Y or Q^2^ (*p* < 0.05; 0/200 for both parameters).

Untargeted LC–MS/MS metabolomics was performed to characterize the metabolic changes associated with SBJ fermentation. As shown in Figure 3a, the PLS-DA score plot showed clear separation among the B1, S4, and YJ2 groups. The final PLS-DA model yielded cumulative R^2^X, R^2^Y, and Q^2^ values of 0.589, 0.967, and 0.903, respectively.

Model robustness was further evaluated using 200 permutation tests. None of the permuted models exceeded the original model for either R^2^Y or Q^2^ (*p* < 0.05; 0/200 for both parameters), supporting the robustness of the observed group discrimination and providing no evidence of substantial model overfitting under this permutation criterion.

As shown in Figure 3a, the PLS-DA score plot clearly separated the B1, S4, and YJ2 groups, indicating that LAB fermentation reshaped the metabolic profile of SBJ in a strain-dependent manner. This is consistent with previous reports showing that LAB metabolism in plant-based matrices is highly strain-dependent and involves coordinated changes in organic acids, carbohydrates, amino acids, and flavor-related metabolites [3,24,25]. The clustering heatmap further confirmed distinct metabolite abundance patterns among the three groups (Figure 3c).

Compared with YJ2, S4 showed a more pronounced decrease in malic acid-related metabolites. Features putatively annotated as malic acid showed markedly lower abundance in the S4 group, accompanied by decreases in citric acid, isocitric acid-related signals, and fumaric acid (Figure 3c). These metabolites are closely associated with the original high-acidity organic acid pool of SBJ, and their decline indicates that S4 fermentation reduced malic acid and other organic acids contributing to sharp acidity. This result supports the phenotypic observation that S4 exhibited stronger malic acid degradation and better acidity modulation than YJ2.

KEGG enrichment analysis further showed that the differential metabolites were mainly associated with central carbon metabolism, the citrate cycle/TCA-related pathway, pyruvate metabolism, glyoxylate and dicarboxylate metabolism, and amino acid metabolism (Figure 3b). These pathways are directly or indirectly linked to malate conversion, citrate utilization, fumarate/succinate interconversion, pyruvate formation, and downstream flavor-related metabolite synthesis. Previous studies have emphasized that LAB adaptation in acidic matrices depends on the coordination of organic acid metabolism, intracellular pH regulation, redox balance, and energy homeostasis [3,26].

Meanwhile, S4 fermentation promoted the accumulation of fermentation-derived metabolites, including succinic acid, 2-oxoglutaric acid, D-3-phenyllactic acid, hydroxyphenyllactic acid, D-octopine, and N-lactoyl-phenylalanine (Figure 3d). This pattern is consistent with broader redistribution of carbon toward pyruvate- and aromatic amino acid-related metabolic branches. LAB fermentation of fruit and vegetable matrices has been reported to remodel organic acids and bioactive metabolites rather than merely reducing acidity [3,24]. Therefore, the increase in phenyllactic acid and hydroxyphenyllactic acid derivatives in S4-fermented SBJ may reflect the conversion of pyruvate- or aromatic amino acid-derived intermediates into milder fermentation-related metabolites. In addition, the accumulation of N-lactoyl-phenylalanine further indicates enhanced lactoyl-related metabolism during fermentation, although this observation should not be considered a substitute for direct quantification of free lactate [27].

Correlation analysis further supported this metabolic transition. Malic acid, citric acid, fumaric acid, and isocitric acid-related metabolites were positively correlated with each other, forming a high-acidity organic acid module (Figure 3e). In contrast, phenyllactic acid derivatives, hydroxyphenyllactic acid derivatives, succinic acid, and N-lactoyl-phenylalanine formed another metabolite module associated with fermentation-derived products. This opposite pattern indicates a shift from the original malic acid-dominated acidic profile toward a metabolite profile enriched in lactate-, pyruvate-, and phenyllactic acid-related products. Overall, the metabolomic results demonstrate that S4 fermentation was accompanied by extensive remodeling of the organic-acid and related metabolic profiles, characterized by decreased abundance of malic acid and several other organic acids together with increased abundance of succinic acid, 2-oxoglutaric acid, and several fermentation-associated metabolites. These abundance changes are consistent with metabolic redistribution but do not establish direct carbon flow from malate into the accumulated products.

### 3.4. Transcriptomic Analysis Reveals Distinct Temporal Strategies of Malate Utilization and Cellular Adaptation

**Figure 4:**
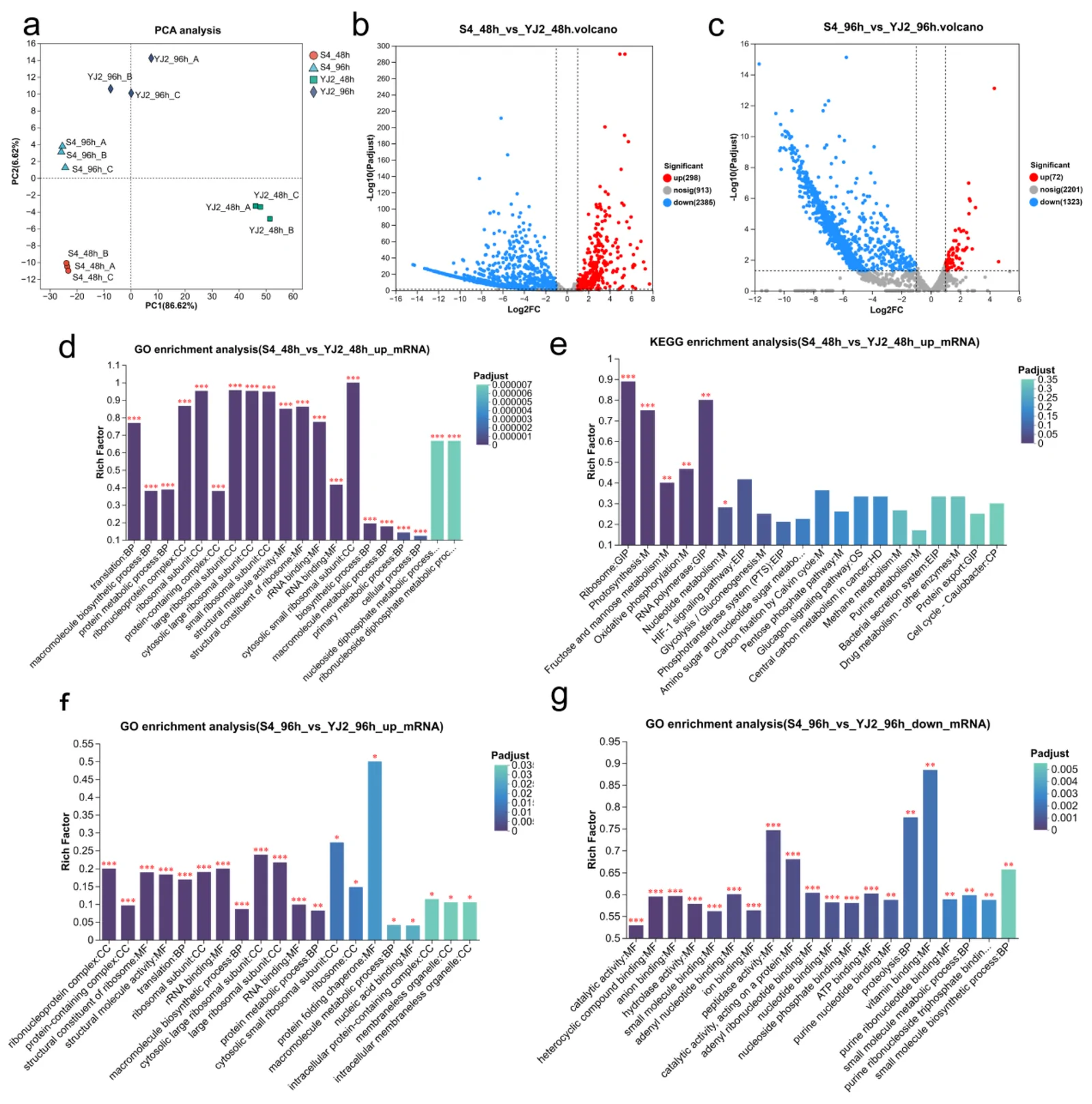
Distinct temporal transcriptomic programs differentiate *Lactiplantibacillus plantarum* S4 from *Lacticaseibacillus paracasei* YJ2 during sea buckthorn juice fermentation. (**a**) Principal component analysis of the global transcriptomic profiles of S4 and YJ2 after 48 and 96 h of fermentation. (**b**,**c**) Volcano plots showing differentially expressed genes between S4 and YJ2 at 48 h (**b**) and 96 h (**c**). (**d**) Gene Ontology enrichment analysis of genes expressed at higher levels in S4 than in YJ2 at 48 h. (**e**) Kyoto Encyclopedia of Genes and Genomes pathway enrichment analysis of genes expressed at higher levels in S4 than in YJ2 at 48 h. (**f**) Gene Ontology enrichment analysis of genes expressed at higher levels in S4 than in YJ2 at 96 h. (**g**) Gene Ontology enrichment analysis of genes expressed at lower levels in S4 than in YJ2 at 96 h. * *p* < 0.05, ** *p* < 0.01, *** *p* < 0.001.

Transcriptomic profiling revealed marked strain- and time-dependent differences between S4 and YJ2 during sea buckthorn juice fermentation. Principal component analysis clearly separated the four experimental groups, with PC1 and PC2 accounting for 86.62% and 6.62% of the total variance, respectively, indicating distinct transcriptional states associated with strain identity and fermentation stage (Figure 4a). At 48 h, 298 genes were expressed at higher levels and 2385 at lower levels in S4 than in YJ2 (Figure 4b). By 96 h, these numbers had decreased to 72 and 1323, respectively, suggesting a more restricted late-stage transcriptional response (Figure 4c).

At 48 h, genes expressed at higher levels in S4 were primarily enriched in translation, ribosomal organization, RNA binding, macromolecule biosynthesis, RNA polymerase activity, oxidative phosphorylation, fructose and mannose metabolism, glycolysis/gluconeogenesis, and nucleotide metabolism (Figure 4d,e). Consistent with these enrichment patterns, the PTS-associated carbohydrate transport genes pts9AB and pts9C were markedly upregulated in S4, with log_2_FC values of 5.351 and 6.241, respectively (Table 4). Glycolytic and pyruvate-metabolism genes, including fba, gpmA2, pgk, pyk, and pdhA, were also expressed at higher levels. This coordinated transcriptional pattern supports enhanced carbohydrate uptake, glycolytic throughput, substrate-level phosphorylation, pyruvate formation, and acetyl-CoA generation. Higher expression of atpE, rpoB, and secY further indicated increased capacity for ATP production, transcription, and protein translocation. Thus, during early fermentation, S4 preferentially invested in carbohydrate assimilation and central carbon metabolism, thereby supplying the energy and metabolic intermediates required for rapid growth, organic-acid conversion, and downstream metabolic remodeling.

Notably, genes directly or indirectly associated with malate utilization, including mleP, mleS, mae, citP, fum, and pycA, showed lower transcript abundance in S4 than in YJ2 at both sampling points (Table 4 and Table 5). This pattern should not be interpreted as evidence of weaker malate utilization by S4. Phenotypic measurements demonstrated substantially faster malate depletion by S4. The crude-extract assay showed higher net malate-conversion activity under MLE-favoring conditions, whereas comparative molecular docking suggested possible strain-dependent differences in the predicted malate-recognition environments of MleP and MleS. These findings are consistent with a functional contribution of the MleP-MleS pathway but do not establish higher MleP transport flux or MleS-specific catalytic activity. Accordingly, the discrepancy between transcript abundance and phenotype likely reflects regulation at multiple levels, and the present data should not be used to infer pathway flux from steady-state transcript abundance alone.

Transcript levels alone are insufficient to predict metabolic flux because pathway activity is additionally controlled by enzyme abundance, catalytic turnover, substrate availability, allosteric regulation, and post-transcriptional processes [28]. Moreover, the malolactic-enzyme and malic-enzyme pathways can be functionally connected while remaining independently regulated in lactic acid bacteria [29]. Given that S4 had already degraded a large proportion of the available malate by 48 h, its lower mleP and mleS transcript levels may reflect attenuation of pathway transcription following an earlier induction phase. In contrast, the higher abundance of these transcripts in YJ2 may represent a substrate-driven or compensatory response to its greater residual malate burden. In *Lacticaseibacillus paracasei*, L-malate can act as a concentration-dependent co-inducer of the mleST operon; therefore, persistent malate availability may sustain elevated pathway transcription without necessarily producing a proportionally higher conversion rate [30]. The interstrain log_2_FC values consequently describe relative transcript abundance at the selected sampling points rather than absolute enzyme capacity or malate-conversion flux.

To distinguish true temporal regulation from changes in the S4-versus-YJ2 contrast, direct within-strain comparisons were performed between 48 and 96 h for both S4 and YJ2 (Table 6). These analyses provide a direct basis for evaluating time-dependent transcriptional remodeling within each strain.

By 96 h, the transcriptional program of S4 had shifted from broad substrate acquisition toward cellular maintenance. The PTS genes pts9AB and pts9C were expressed at lower levels in S4, whereas gpmA2, atpE, rpoB, rpoC, dnaK, and hrcA remained moderately elevated (Table 3). The enrichment of genes associated with translation, ribosomal organization, RNA binding, and protein folding among S4-high genes (Figure 4f) suggests that S4 maintained translational competence, energy homeostasis, and proteostasis after extensive malate depletion. In particular, increased expression of dnaK and hrcA is consistent with their established roles in preventing protein misfolding and coordinating the acid-stress response of *L. plantarum* [15,16].

Conversely, clpB, prsA2, dltB, and gadB were expressed at substantially lower levels in S4 than in YJ2. Together with the enrichment of catalytic, hydrolase, peptidase, proteolytic, ATP-binding, and small-molecule metabolic functions among S4-low genes (Figure 4g), these results indicate that YJ2 retained a broader late-stage catabolic and stress-response program. The elevated expression of glutamate decarboxylation, cell-envelope modification, protein disaggregation, and extracellular protein-folding functions in YJ2 likely reflects continued adaptation to residual organic-acid pressure. In contrast, S4 exhibited a more selective quality-control response, consistent with the earlier resolution of malate-associated acid stress.

**Figure 5:**
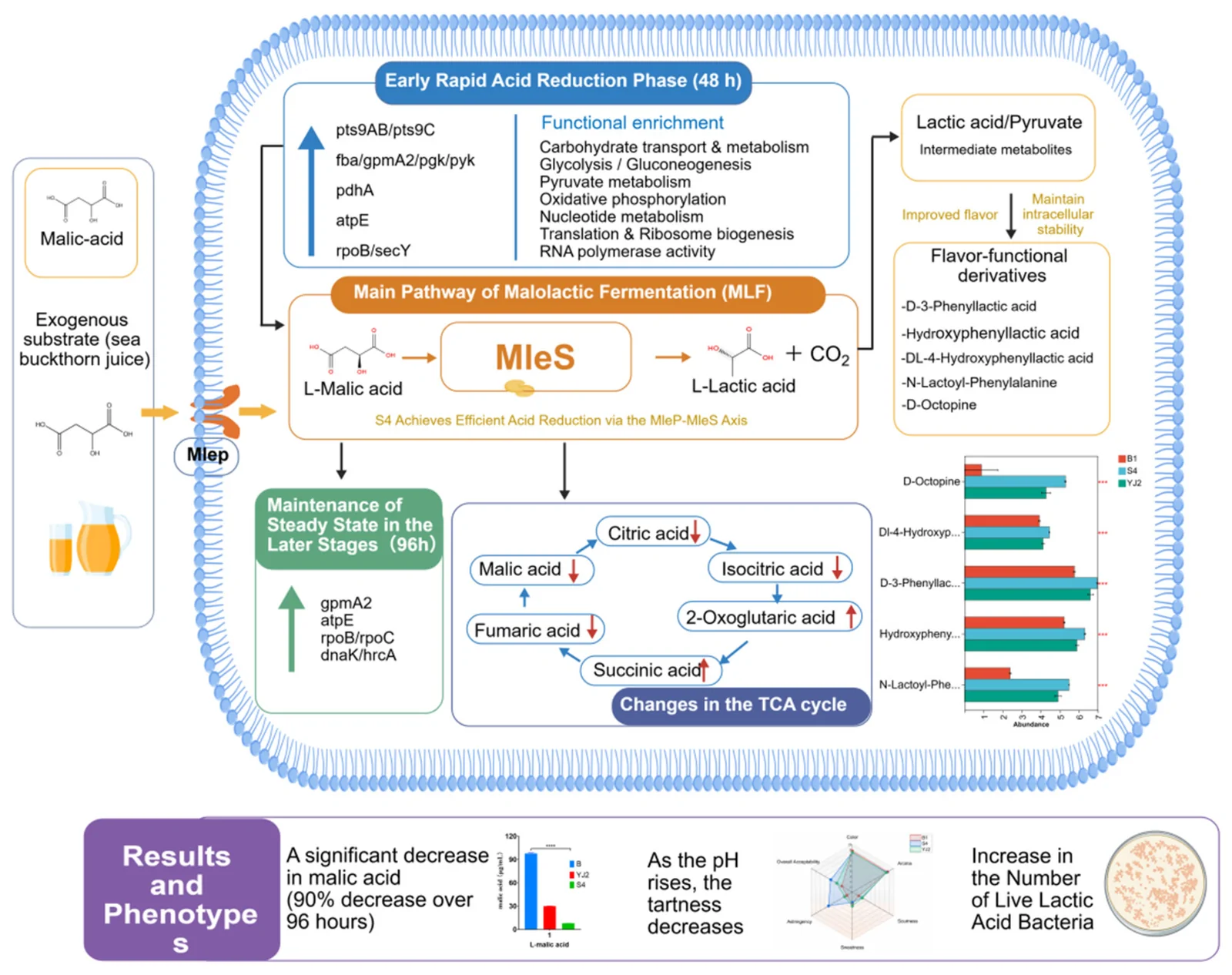
Proposed working model of malate-associated metabolic responses in *Lactiplantibacillus plantarum* S4 during SBJ fermentation. Created with BioGDP.com [31]. The placement of MleP as a malate transporter and MleS as a malolactic enzyme reflects the proposed pathway model. Molecular docking was used only to explore possible differences in malate recognition and was not used to infer MleP transport rate or MleS catalytic efficiency. *** *p* < 0.001, **** *p* < 0.0001.

Integrating fermentation phenotypes, molecular docking, metabolomic and transcriptomic datasets supports a stage-dependent working model underlying the efficient malic acid degradation by *Lactiplantibacillus plantarum* S4 (Figure 5).

During the early rapid acid-reduction phase at 48 h, the model positions MleP as the malate transporter and MleS as the malolactic enzyme that converts intracellular L-malic acid to L-lactic acid and CO_2_. In this study, S4 exhibited higher crude-extract malate-conversion activity under MLE-favoring conditions, while comparative molecular docking suggested potential differences in malate recognition between the S4- and YJ2-derived MleP and MleS models. As docking analysis cannot measure transport kinetics or catalytic turnover, these structural observations are regarded as supportive, rather than mechanistic evidence for the involvement of the MleP–MleS system.

Concurrently, upregulation of pts9AB and pts9C indicated enhanced PTS-dependent carbohydrate uptake, and elevated expression of fba, gpmA2, pgk and pyk was consistent with increased glycolytic capacity. Upregulation of pdhA, atpE, rpoB and secY further suggested enhanced pyruvate conversion, ATP synthesis, transcriptional capacity and protein export. These transcriptional responses were consistent with the enrichment of carbohydrate metabolism, glycolysis/gluconeogenesis, pyruvate metabolism, oxidative phosphorylation, nucleotide metabolism, ribosome biogenesis and RNA polymerase activity.

Metabolomic changes further demonstrated that S4 remodeled the broader organic-acid network rather than merely depleting malate. Malic acid, citric acid, isocitric acid-related metabolites, and fumaric acid decreased, whereas succinic acid and 2-oxoglutaric acid increased, a pattern consistent with extensive remodeling of TCA-related and dicarboxylate-associated metabolism. This metabolic remodeling was accompanied by increased abundance of phenyllactic acid-related metabolites, hydroxyphenyllactic acid-related metabolites, octopine-related signals, and N-lactoyl-phenylalanine.

By 96 h, S4 shifted from rapid substrate utilization toward cellular maintenance. Sustained expression of gpmA2, atpE, rpoB/rpoC, dnaK, and hrcA indicated continued support for glycolytic competence, energy homeostasis, transcription, protein folding, and acid-stress adaptation. This temporally coordinated strategy was consistent with the observed 91.98% reduction in malic acid, increased pH, decreased sourness and astringency, improved overall acceptability, and higher viable-cell counts. Overall, the data support a model in which MleP-MleS-mediated malate conversion, together with central-carbon redistribution, organic-acid remodeling, flavor-metabolite formation, and late-stage homeostatic maintenance, contributes to the superior deacidification performance of S4; however, the relative MleP transport capacity remains inferential.

## 4. Conclusions

In the present comparison of two LAB isolates, *Lactiplantibacillus plantarum* S4 removed 91.98% of malic acid from SBJ within 96 h, compared with 69.50% for *Lacticaseibacillus paracasei* YJ2, and showed higher viable-cell counts and overall sensory acceptability under the tested conditions. These results demonstrate a clear phenotypic difference between S4 and YJ2 but, because only one isolate of each species was examined, they do not distinguish species-level effects from isolate-specific characteristics and should not be generalized to *L. plantarum* and *L. paracasei* as species.

The integrated enzyme, molecular docking, metabolomic, and transcriptomic data are consistent with a contribution of the MleP–MleS-associated malate-conversion system to the greater malate-removal phenotype of S4. However, MleP-mediated transport, MleS-specific catalytic activity, and stoichiometric lactate production were not directly quantified; therefore, the relative contribution and flux of this pathway remain inferential. The greater malate depletion observed for S4 was also associated with differences in central-carbon metabolism, organic-acid remodeling, fermentation-related metabolite profiles, and late-stage cellular maintenance. These observations support a proposed working model linking malate-associated conversion with broader metabolic and stress-adaptation responses rather than establishing a definitive causal mechanism.

Overall, the results identify S4 as a promising candidate for further development as an SBJ starter culture. Validation across a broader collection of *L. plantarum*, *L. paracasei*, and other LAB strains, together with direct measurements of malate transport, enzyme-specific activity, and lactate formation, will be required to determine the generality and causal basis of the observed phenotype.

## Figures and Tables

**Table 1:** Criteria and scoring system for sensory evaluation of sea buckthorn juice.

Sensory Attribute	Evaluation Criteria	Score
Color (10 points)	Bright, uniform color with good gloss	8–10
	Relatively bright and uniform color, with no obvious browning	5–7
	Dull and non-uniform color, with severe browning	0–4
Aroma (10 points)	Pronounced fruity aroma with no off-odor	8–10
	Weak fruity aroma with no obvious off-odor	5–7
	No perceptible fruity aroma, with an obvious off-odor	0–4
Acidity (10 points)	Mild and well-balanced acidity, harmonious with the characteristic sea buckthorn aroma	8–10
	Moderate acidity without a strong irritating sensation	5–7
	Harsh and strongly irritating sourness	0–4
Sweetness (10 points)	Pleasant and clean sweetness, perceived promptly and well integrated with other taste sensations, resulting in a complex taste profile	8–10
	Mild sweetness with relatively short persistence	5–7
	No obvious sweetness upon tasting, with weak perceived sweetness	0–4
Astringency (10 points)	No perceptible astringency, with a smooth mouthfeel	8–10
	Slight astringency without obvious oral roughness	5–7
	Strong astringency accompanied by a pronounced puckering sensation	0–4
Overall acceptability (10 points)	Pleasant aroma and taste, with a high willingness to taste repeatedly	8–10
	Neither clearly liked nor disliked; generally acceptable for tasting	5–7
	Disliked, with a clear sense of aversion	0–4

Note: Each sensory attribute was evaluated on a 10-point quality scale, with higher scores indicating more desirable sensory characteristics rather than greater sensory intensity.

**Table 2:** SWISS-MODEL templates and quality metrics of malate-associated protein models used for molecular docking.

Strain	Protein	Length (aa)	Template	Sequence Identity (%)	Coverage	GMQE	Predicted Oligomeric State
S4	MAE	386	A0A829WQ12.1.A	99.22	1.00	0.96	Monomer
YJ2	MAE	392	2a9f.1.A	73.64	1.00	0.83	Homo-dimer
S4	MDH	362	A0A2S3U3H1.1.A	99.45	1.00	0.96	Monomer
YJ2	MDH	392	A0A806E8Z5.1.A	99.49	1.00	0.96	Monomer
S4	MleP	323	A0A0C3A115.1.A	78.33	1.00	0.90	Monomer
YJ2	MleP	315	C2FDQ6.1.A	99.37	1.00	0.90	Monomer
S4	MleS	557	A0A809KC57.1.A	81.89	1.00	0.94	Monomer
YJ2	MleS	555	S2RRP4.1.A	100.00	1.00	0.95	Monomer

**Table 3:** Comparison of binding energies between malate-related proteins/enzymes and malic acid in molecular docking.

Protein	S4 (kcal/mol)	YJ2 (kcal/mol)	S4 Mean	YJ2 Mean
MleP	−5.4 to −4.6	−4.2 to −3.7	−4.97	−3.91
MleS	−5.6 to −5.1	−4.7 to −4.3	−5.28	−4.50
MAE	−4.6 to −4.3	−4.4 to −4.0	−4.41	−4.18
MDH	−4.6 to −3.9	−4.5 to −4.0	−4.09	−4.26

**Table 4:** Representative differentially expressed genes between S4 and YJ2 at 48 h.

Gene	Functional Category	log2FC (S4/YJ2)	Expression Pattern
mleP	Malate transport	−8.250	Down
mleS	Malolactic fermentation	−4.171	Down
mae	NAD-dependent malate decarboxylation	−8.156	Down
pts9AB	PTS-dependent carbohydrate uptake	5.351	Up
pts9C	PTS-dependent carbohydrate uptake	6.241	Up
fba	Glycolysis	3.590	Up
gpmA2	Glycolysis	3.412	Up
pgi	Glycolysis	3.356	Up
pgk	Glycolysis and ATP generation	2.632	Up
pyk	Pyruvate formation	2.468	Up
pdhA	Pyruvate-to-acetyl-CoA conversion	2.427	Up
atpE	ATP synthesis and proton homeostasis	2.695	Up
rpoB	Transcriptional capacity	2.938	Up
secY	Protein export	3.547	Up
npr2	Oxidative-stress protection	2.068	Up
trxH	Thiol redox homeostasis	1.288	Up
clpB	Protein disaggregation and stress response	−10.582	Down
gadB	Glutamate decarboxylation and acid resistance	−11.559	Down

Note: log2FC was calculated as S4/YJ2. Positive values and “Up” indicate higher expression in S4, whereas negative values and “Down” indicate higher expression in YJ2. All listed genes were significantly differentially expressed in the corresponding dataset.

**Table 5:** Representative differentially expressed genes between S4 and YJ2 at 96 h.

Gene	Functional Category	log2FC (S4/YJ2)	Expression Pattern
mleP	Malate transport	−4.832	Down
mleS	Malolactic fermentation	−3.864	Down
mae	NAD-dependent malate decarboxylation	−5.093	Down
pts9AB	PTS-dependent carbohydrate uptake	−2.958	Down
pts9C	PTS-dependent carbohydrate uptake	−2.339	Down
gpmA2	Glycolysis	1.162	Up
atpE	ATP synthesis and proton homeostasis	1.534	Up
rpoB	Transcriptional capacity	1.154	Up
rpoC	Transcriptional capacity	1.191	Up
dnaK	Protein folding and quality control	1.777	Up
hrcA	Heat-shock response regulation	2.673	Up
clpB	Protein disaggregation and stress response	−8.393	Down
prsA2	Extracellular protein folding	−8.470	Down
dltB	Cell-wall D-alanylation	−9.461	Down
gadB	Glutamate decarboxylation and acid resistance	−8.863	Down

**Table 6:** Key time-dependent differentially expressed genes associated with malate metabolism and cellular homeostasis in S4 and YJ2.

Gene	Functional Category	S4 log2FC (48 h/96 h)	S4 Expression Pattern	YJ2 log2FC (48 h/96 h)	YJ2 Expression Pattern
mleP	Malate transport	4.398	Up	1.251	Up
mleS	Malolactic fermentation	1.086	Up	NR	NR
pts9AB	PTS-dependent carbohydrate uptake	4.845	Up	−5.643	Down
pts9C	PTS-dependent carbohydrate uptake	4.652	Up	−6.129	Down
pts9D	PTS-dependent carbohydrate uptake	3.448	Up	−7.158	Down
pgi	Glycolysis	3.016	Up	−1.744	Down
pgk	Glycolysis and ATP generation	2.094	Up	NR	NR
ldh1	Lactate-pyruvate metabolism	1.570	Up	−1.790	Down
pdhA	Pyruvate-to-acetyl-CoA metabolism	2.337	Up	−2.369	Down
pdhB	Pyruvate-to-acetyl-CoA metabolism	1.973	Up	NR	NR
pox3	Pyruvate oxidation pathway	2.304	Up	−1.666	Down
npr2	Oxidative-stress protection	3.499	Up	−1.601	Down
trxH	Thiol redox homeostasis	2.398	Up	NR	NR
ftsA	Cell division	2.591	Up	NR	NR
dnaK	Molecular chaperone and protein folding	−2.853	Down	NR	NR
dnaJ	Molecular chaperone and protein folding	−2.099	Down	−3.266	Down
groL	Molecular chaperone and protein folding	−1.881	Down	NR	NR
groS	Molecular chaperone and protein folding	−1.868	Down	NR	NR
atpD	Energy and proton homeostasis	−1.279	Down	−5.137	Down
opuA	Compatible-solute transport/Osmoprotection	−1.806	Down	−4.995	Down
dltC1	Cell-envelope homeostasis	−2.319	Down	−3.403	Down
clpX	Protein quality control	−3.530	Down	−5.023	Down
clpE	Protein quality control	−2.463	Down	NR	NR
lrgB	Regulation of cell-wall hydrolysis	−2.800	Down	NR	NR
hrcA	Heat-shock response regulation	−5.256	Down	NR	NR
mdh	Malate-oxaloacetate metabolism	NR	NR	1.943	Up
fba	Glycolysis	NR	NR	−4.347	Down
gltT	Ion and amino-acid transport	NR	NR	−2.581	Down
dltX	Cell-envelope remodeling	NR	NR	−3.702	Down
trxB	Thiol redox homeostasis	NR	NR	−2.038	Down

Note: log2FC was calculated as 48 h/96 h. Positive values and “Up” indicate higher expression in 48 h, whereas negative values and “Down” indicate higher expression in 96 h. All listed genes were significantly differentially expressed in the corresponding dataset.

## Data Availability

The original contributions presented in this study are included in the article. Further inquiries can be directed to the corresponding author.
